# CD2-targeted nanoparticles encapsulating IL-2 induce tolerogenic Tregs and TGF-β-producing NK cells that stabilize Tregs for long-term therapeutic efficacy in immune-mediated disorders

**DOI:** 10.3389/fimmu.2025.1587237

**Published:** 2025-07-29

**Authors:** David A. Horwitz, Dongin Kim, Chang Kang, Katja Brion, Sean Bickerton, Antonio La Cava

**Affiliations:** ^1^ General Nanotherapeutics, Santa Monica, CA, United States; ^2^ Keck School of Medicine, University of Southern California, Los Angeles, CA, United States; ^3^ Department of Pharmaceutical Sciences, University of Oklahoma Health Sciences Center, Oklahoma City, OK, United States; ^4^ Department of Medicine, University of California, Los Angeles, Los Angeles, CA, United States; ^5^ Department of Biomedical Engineering, Yale University, New Haven, CT, United States; ^6^ Department of Medicina Molecolare e Biotecnologie Mediche, Federico II University of Naples, Naples, Italy

**Keywords:** nanoparticles, T regulatory cells (Tregs), NK cells, autoimmunity, immune tolerance, IL-2, TGF-β

## Abstract

T regulatory cells (Tregs) generated in the periphery (pTregs) are initially unstable, but some of them stabilize with time. The stabilization signals, however, are poorly understood. We have previously reported that the treatment of mice with poly(lactic-co-glycolic) acid (PLGA) nanoparticles (NPs) decorated with anti-CD2 antibodies and encapsulating IL-2 and TGF-β induced tolerogenic CD4^+^ and CD8^+^ pTregs that protected mice from fatal graft-versus-host disease (GvHD). These NPs also induced TGF-β-producing NK cells. Here we show that initially unstable Tregs are stabilized and maintained by NK-cell derived TGF-β. Blockade of TGF-β signaling or NK cell depletion hindered the induction of Tregs and converted tolerogenic responses into immunogenic responses, leading to an accelerated demise of the mice. IL-2 from the NPs and TGF-β from NP-induced NK cells were sufficient for the maintenance of the Tregs, making the encapsulated TGF-β unnecessary. These results identify a new non-redundant cellular source of TGF-β required for the support of newly induced Tregs. NPs inducing cross-communicating innate and adaptive tolerogenic cells can represent a new cell-targeted approach to induce and maintain long-term immune tolerance in immune-mediated diseases.

## 1 Introduction

Regulatory T cells (Tregs) play a vital role in modulating immune responses to self-antigens and foreign antigens. CD4^+^ T cells that express the transcription factor FOXP3 are most important in this process (1).

CD4^+^CD25^+^FOXP3^+^ Tregs can be divided into two major groups, those generated in the thymus (tTregs), and those induced from conventional CD4^+^ T cells. The latter are named pTregs when generated in the periphery *in vivo*, and iTregs when generated *in vitro* (2). While tTregs are functionally stable, pTregs induced from conventional T cells are typically unstable, and the local presence of inflammatory cytokines can convert them into non-regulatory effector cells (3).

The instability of pTregs associated with their susceptibility to lose FOXP3 expression in the Treg-specific demethylation regions (TSDR) in the *FOXP3* gene region (4). By contrast, demethylated CpG islands in the *FOXP3* TSDR contribute to tTregs stability (5). Interestingly, for unexplained reasons, newly generated pTregs may become stable within a few weeks and help preventing immune-mediated diseases (6, 7).

One contributor to acquiring Treg stability is the cytokine TGF-β (8). When combined with IL-2 and continuous T cell receptor (TCR) stimulation, this cytokine is required for induction, maintenance, and survival of Tregs (9, 10). Since IL-2 and TGF-β production and/or signaling can be deficient in autoimmune diseases including SLE (11, 12), rheumatoid arthritis (13, 14), type 1 diabetes (15, 16), multiple sclerosis (17, 18) and inflammatory bowel disease (19, 20), these abnormalities can contribute to Treg instability. In these diseases, correction of the IL-2 and TGF-β unbalances could have therapeutic potential.

IL-2 plays a major role in stabilizing Tregs (21, 22). However, clinical trials with low-dose IL-2 to expand functional Tregs in GvHD and autoimmune diseases have given mixed results (23–27). Moreover, clinical trials in SLE with IL-2 muteins engineered to react preferentially with Tregs have not been succesful (28).

Here we used anti-CD2 antibodies to decorate NPs because anti-CD2 antibodies induce NK cells to produce TGF-β (29). Unlike anti-CD3 antibodies, anti-CD2 antibodies do not stimulate production of IgM or IgG because they induce NK cells to produce TGF-β, which suppresses antibody production (30).

The results show that Tegs require a synergistic activity of TGF-β and IL-2. The tolerogenic effects of IL-2 are dependent on TGF-β and that an NK cell subset provides non-redundant TGF-β for Tregs. We have, therefore, identified a link between innate and adaptive immune cells. Even in a strong proinflammatory environment of a disease such as GVHD, the TGF-β from tolerogenic NK cells can stabilize Tregs and sustain long-term function. These findings suggest that some failures of IL-2 based clinical trials could be due to insufficient TGF-β.

## 2 Materials and methods

### 2.1 Preparation of nanoparticles

**Figure 1:**
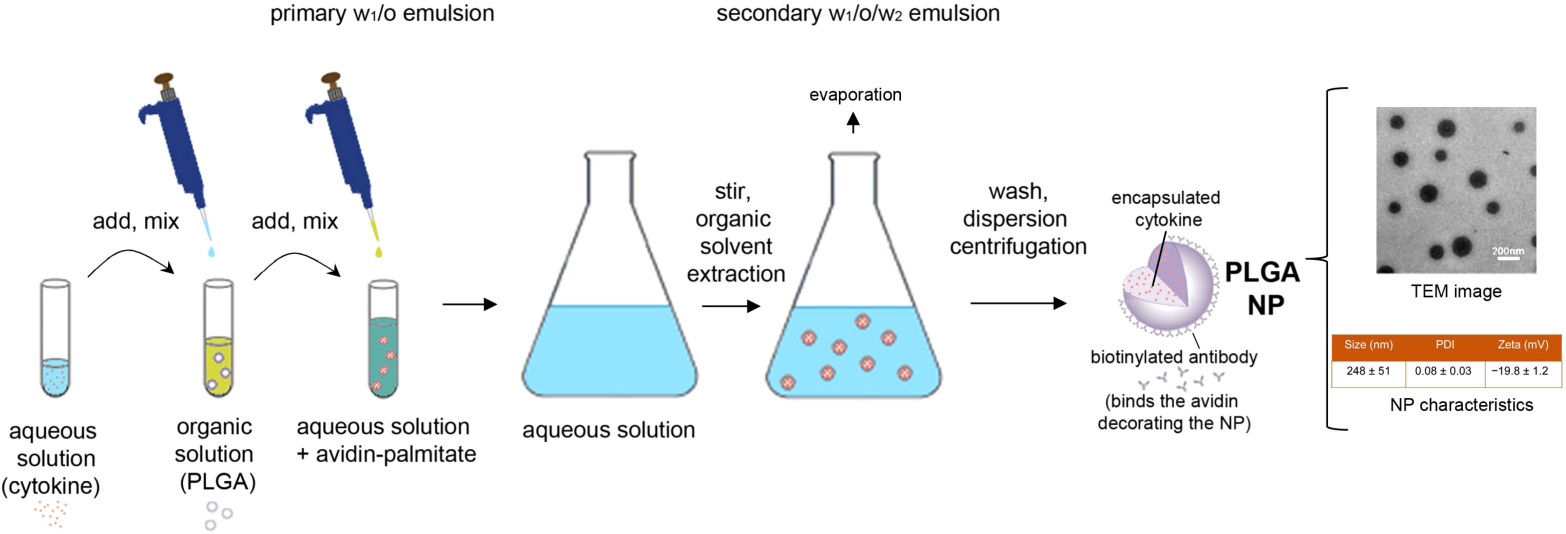
Schematic representation of the preparation and characteristics of the nanoparticles used in the study. PLGA, poly(lactic-co-glycolic) acid; NP, nanoparticle; TEM, transmission electron microscopy; PDI, polydispersity index.

Poly lactic-co-glycolic acid (PLGA) NPs were prepared as described (31), using a validated protocol shown in **Figure 1**. After preparation, the NPs were characterized with standard procedures for physical properties, encapsulation metrics and release kinetics (32). Dynamic light scattering showed a NP mean ± SD hydrodynamic diameter of 248 ± 51 nm with a low polydispersity index indicative of a uniform NP population with a tight size distribution (**Figure 1**). Cytokine encapsulation was assessed by ELISA after NPs were disrupted using DMSO. Standard curves were generated using cytokine standards with wells supplemented to contain 5% v/v DMSO and the appropriate concentration of empty NPs (33). The NPs contained a mean ± SD of 11 ± 2 ng TGF-β and 2.4 ± 1.2 ng IL‐2 per mg of NP. For cell targeting, NPs diluted in PBS were incubated 10 minutes prior to coupling with biotinylated anti-CD2 antibody (clone RPA-2.10, ThermoFisher Scientific) at a concentration of 2 μg antibody/mg NP.

### 2.2 Human peripheral blood mononuclear cells

Human PBMCs were isolated following Ficoll-Hypaque density gradient centrifugation of heparinized venous blood from healthy adult volunteers under approved institutional IRB protocols. PBMCs were used unseparated or depleted of NK cells using anti-human CD56 microbeads (Miltenyi Biotec) or depleted of NKT cells using biotinylated anti-TCR Va24Jα18 mAb (clone 6B11) (ThermoFisher Scientific) plus streptavidin microbeads (Miltenyi Biotec) with a Miltenyi Biotec autoMACS Pro Cell Separator. Depletion efficiency was in both cases >94%, as assessed by staining with APC-labeled anti-NKP46 mAb (clone 9E2) (ThermoFisher Scientific) for NK cells and anti-TCR Va24Jα18 mAb clone C15 (BD Biosciences) for NKT cells.

### 2.3 Cell cultures

Human PBMCs at a concentration of 2 x 10^6^ cells/ml in RPMI medium supplemented with 2% human serum (Millipore Sigma), 2 mM glutamine, 100 U/mL penicillin and 100 μg/mL streptomycin were cultured for five days at 37°C/5% CO_2_ with 100 μg/mL NPs before harvest for flow cytometry.

### 2.4 Flow cytometry

PBMCs were stained following standard procedures with FITC-, PE-, PerCP- or APC-conjugated anti-human monoclonal antibodies to CD4 (clone RPA-T4), CD8 (RPA-T8), CD25 (MEM-181), CD127 (eBioRDR5), CD3 (OKT3), CD16 (3G8), CD56 (MEM-188), CD19 (HIB19), CD14 (61D3), CD11c (3.9), GARP (G14D9), CD160 (BY55), CD335/NKP46 (9E2), NKG2D (1D11), PD-1 (MIH4), TIM-3 (F38-2E2) and intracellular FoxP3 (PCH101), TGF-β (TB21), or isotype controls. All antibodies were from ThermoFisher Scientific. Data were acquired on a FACSCalibur™ flow cytometer (BD Biosciences, San Jose, CA) and analyzed using BD FACSDiva™ or FlowJo™ software (BD Biosciences).

### 2.5 Mice

Human PBMCs purified as above were transferred into NOD-*scid* IL-2 receptor common γ chain *null* (NOD*-scid Il2rg^tm1Wjl^*) (NSG) mice, to induce human-anti-mouse GvHD (34). NSG mice purchased from the Jackson Laboratory (Bar Harbor, ME) were housed under pathogen-free conditions in microisolator cages with *ad libitum* access to autoclaved food and sterile water. Briefly, 10^7^ fresh human PBMCs were resuspended in 200 µl of PBS in insulin syringes and injected i.v. *via* the tail vein into individual unconditioned NSG mice of 8–12 weeks of age. The mice also received i.p. (individually) 1.5 mg IL-2/TGF-β-encapsulated NPs or IL-2 encapsulated NPs decorated with anti-CD2 antibodies (29), starting on the day of transfer of human PBMCs, according to the protocol of administration at days 0, 3, 6, 9, 12 (29, 34). Parallel groups of mice also received i.p. contralaterally the ALK5 inhibitor SB431542 (Biotechne) at 1 mg/kg/d every other day until 24 days after PBMC transfer. Control mice received empty NPs under identical conditions. The experiments were performed according to guidelines of the Institutional Animal Committee of the University of California Los Angeles. Peripheral blood was taken at serial time points for *ex vivo* immune cell monitoring by flow cytometry. The results show data representative of three or more repeat experiments.

### 2.6 Statistical analyses

Assessment for normal distribution was done by the Shapiro-Wilks test. Comparisons between two groups were evaluated using (*post-hoc*) Student’s *t* test. Comparisons among multiple groups used one-way ANOVA with Bonferroni’s correction. Differences in Kaplan-Meier survival curves were analyzed by the log-rank test. Statistical data analysis was done using Prism software (GraphPad, La Jolla, CA). *P* values <0.05 were considered significant.

## 3 Results

We used an established mouse model of immune-mediated disease characterized by strong inflammation, GvHD, which develops following transfer of human PBMCs into immunodeficient NSG mice (34).

**Figure 2:**
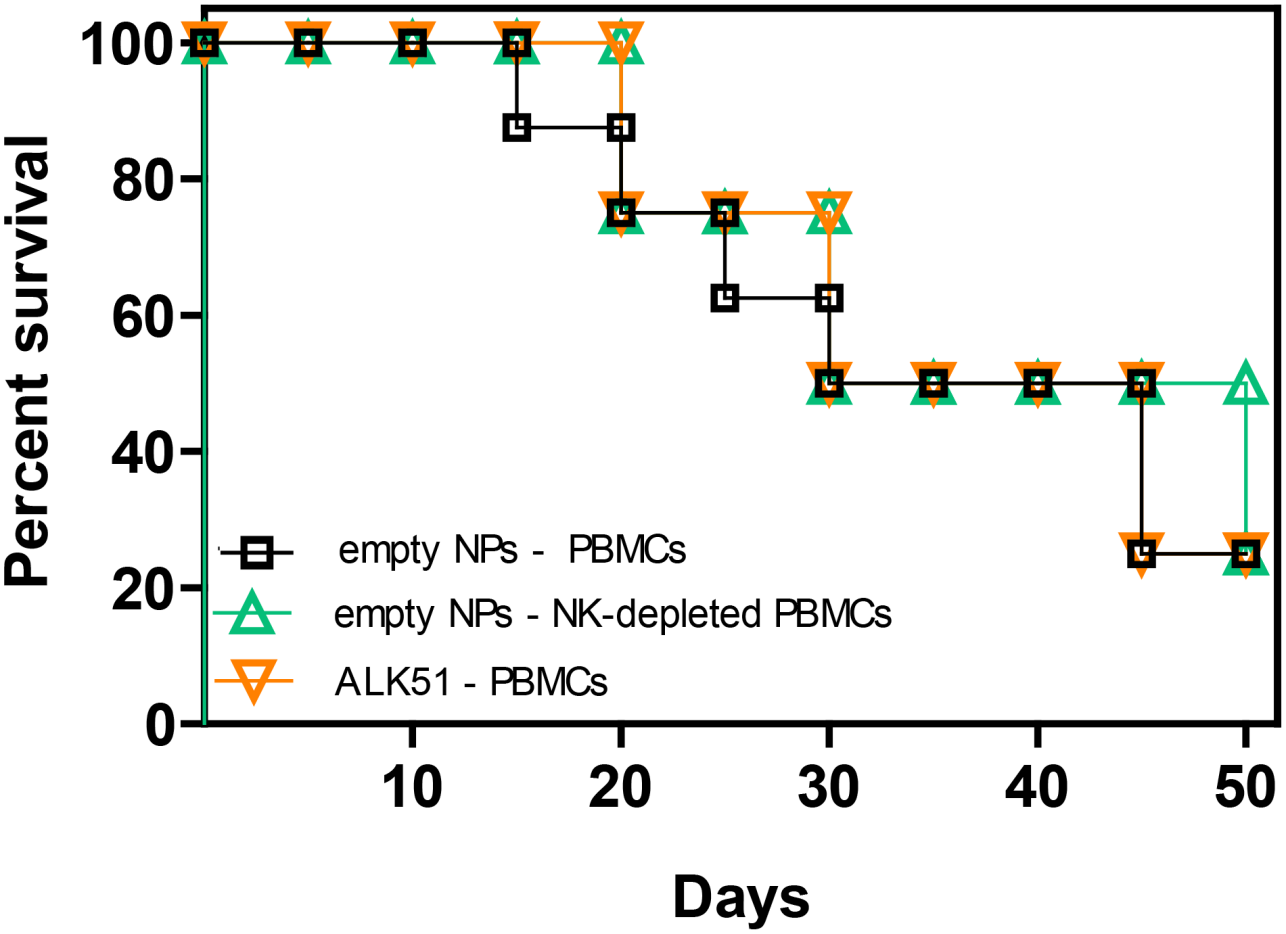
Neither depletion of NK cells nor blockade of TGF-β signaling alter the course of GvHD in NSG mice transferred with xenogeneic human PBMCs. NSG mice (n = 8/group) received on day 0 either 10^7^ unseparated human PBMCs only or together with the TGF-β signaling inhibitor ALK5i. Another group of mice received 10^7^ NK cell-depleted human PBMCs. The PBMCs were from healthy donors. *P* not significant in any comparison.

**Figure 3:**
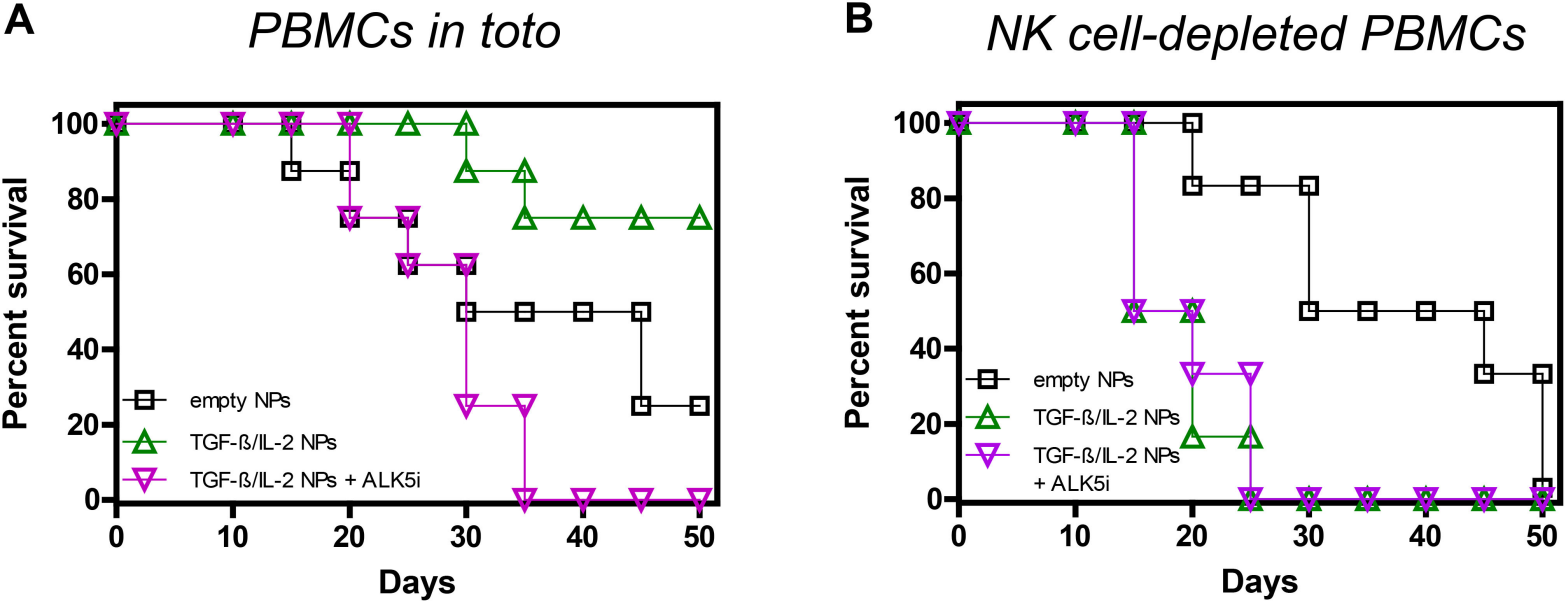
Role of TGF-β and NK cells in the NP-mediated protection of NSG mice from GvHD. Survival curves of NSG mice receiving either 10^7^ unseparated human PBMCs **(A)** (n = 8/group) or 10^7^ NK cell-depleted PBMCs **(B)** (n = 6/group), together with empty NPs or TGF-β/IL-2-encapsulated NPs with or without the TGF-β signaling inhibitor ALK5i. Individual mice received PBMCs from individual healthy donors. **(A)** Empty NPs vs. TGF-β/IL-2 NPs, *P <*0.0001; vs. TGF-β/IL-2 + ALK5i, *P* 0.01. **(B)** Empty NPs vs. TGF-β/IL-2 NPs or TGF-β/IL-2 NPs + ALK5i, *P*<0.0001.

We had previously shown that tolerogenic NPs that inhibit immune responses to specific antigens can protect NSG mice from fatal GvHD by inducing NK cells and TGF-β (34). However, it was not known whether NK cells or TGF-β influenced the GvHD independently of the NPs. In other words, it remained to be addressed whether the NPs were necessary to promote the activity of NK cells and/or TGF-β from a basal state or whether the NPs were dispensable. **Figure 2** shows that neither the blockade of TGF-β signaling following administration of ALK5i nor the depletion of NK cells from human PBMCs before cell transfer altered the course of GvHD as compared with control mice. On the other hand, a 15-day course of treatment with NPs encapsulating TGF-β and IL-2 provided sustained protective effects, since 75% of the mice in the NP-treated group were alive by day 50 vs. 25% of the control group treated with empty NPs (**Figure 3A**).

When TGF-β was inhibited *in vivo*, not only was protection abolished, but the demise of the mice was accelerated. While at day thirty-five 50% of the control mice were alive, all mice succumbed when TGF-β signaling was blocked in NP-treated mice (**Figure 3A**). Thus, without TGF-β in the NPs, the effects of IL-2 were abrogated, suggesting a dependency on TGF-β for the IL-2 tolerogenic effects.

Depletion of NK cells also abolished the protective effects of the NPs, greatly decreasing mice survival (**Figure 3B**). This suggested a contributing role of NK cells to the tolerogenic effects of TGF-β, likely through a common inhibitory mechanism, considering that NK cell depletion coupled with TGF-β inhibition did not further accelerate mortality.

**Figure 4:**
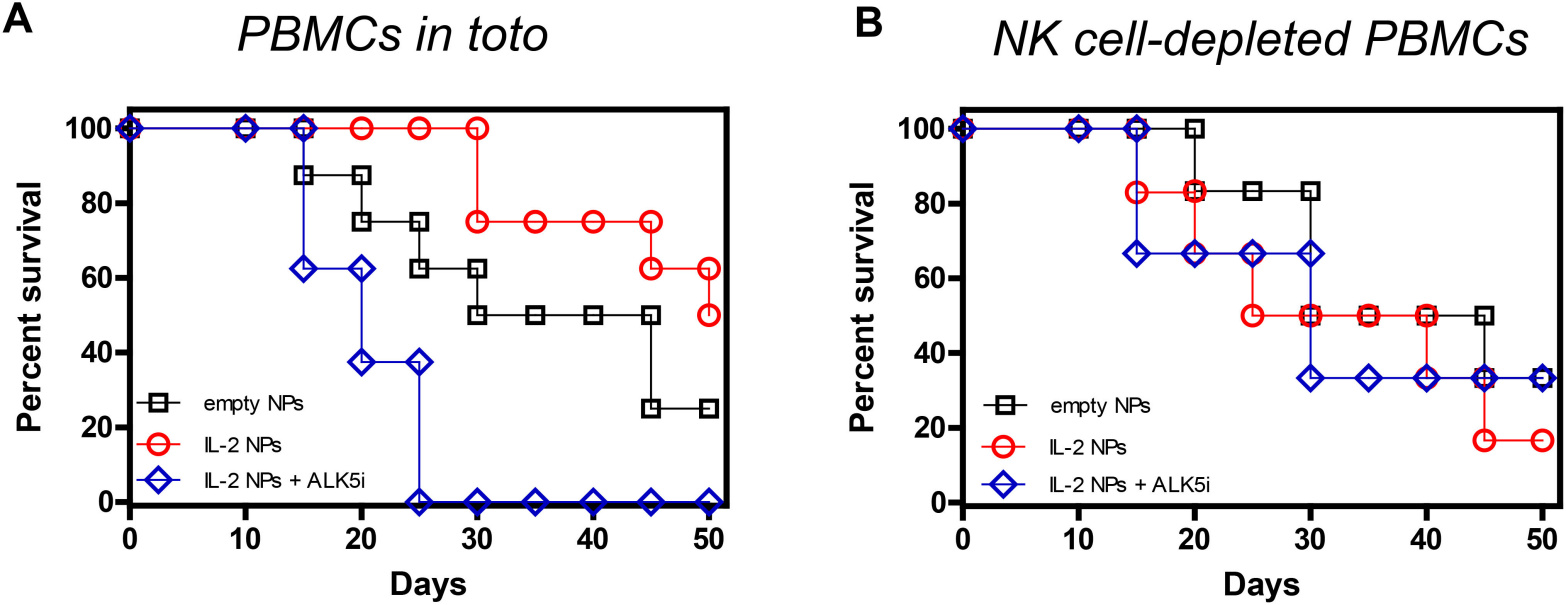
Effects of IL-2 in the NP-mediated protection of NSG mice from GvHD. Survival curves of NSG mice that received either 10^7^ unseparated human PBMCs **(A)** (n = 8/group) or 10^7^ NK cell-depleted PBMCs **(B)** (n = 6/group) together with empty NPs or IL-2-encapsulated NPs alone or together with the TGF-β signaling inhibitor ALK5i. Individual mice received PBMCs from individual healthy donors. **(A)** Empty NPs vs. IL-2 NPs or IL-2 NPs + ALK5i, *P*<0.0001. **(B)** Empty NPs vs. IL-2 NPs, *P*<0.001; vs IL-2 NPs + ALK5i, *P*<0.0001.

If the interpretation of these findings was that NK cells were the primary source of the TGF-β required to protect from GvHD, NPs containing only IL-2 should be sufficient for the protection. **Figure 4A** shows that NPs containing only IL-2 had almost equivalent protective effects as those containing both IL-2 and TGF-β, with half of the mice still alive on day 50. TGF-β dependency was confirmed by the finding that blockade of TGF-β signaling with ALK5i resulted in deleterious effects analogous to those seen in **Figure 3**. Although NK cell depletion also abolished the protective effects of the NPs encapsulating only IL-2 (**Figure 4B**), it did not have the same detrimental effects when TGF-β signaling was blocked in intact PBMCs, i.e., not depleted of NK cells (**Figure 4A**). These results suggest that when TGF-β signaling was blocked, IL-2 from the NPs promoted immunogenic responses instead of tolerogenic responses. Depletion of NK cells abolished these effects, in contrast with what seen after depletion of NK cells in mice receiving NPs encapsulating IL-2 and TGF-β (**Figure 3**).

**Figure 5:**
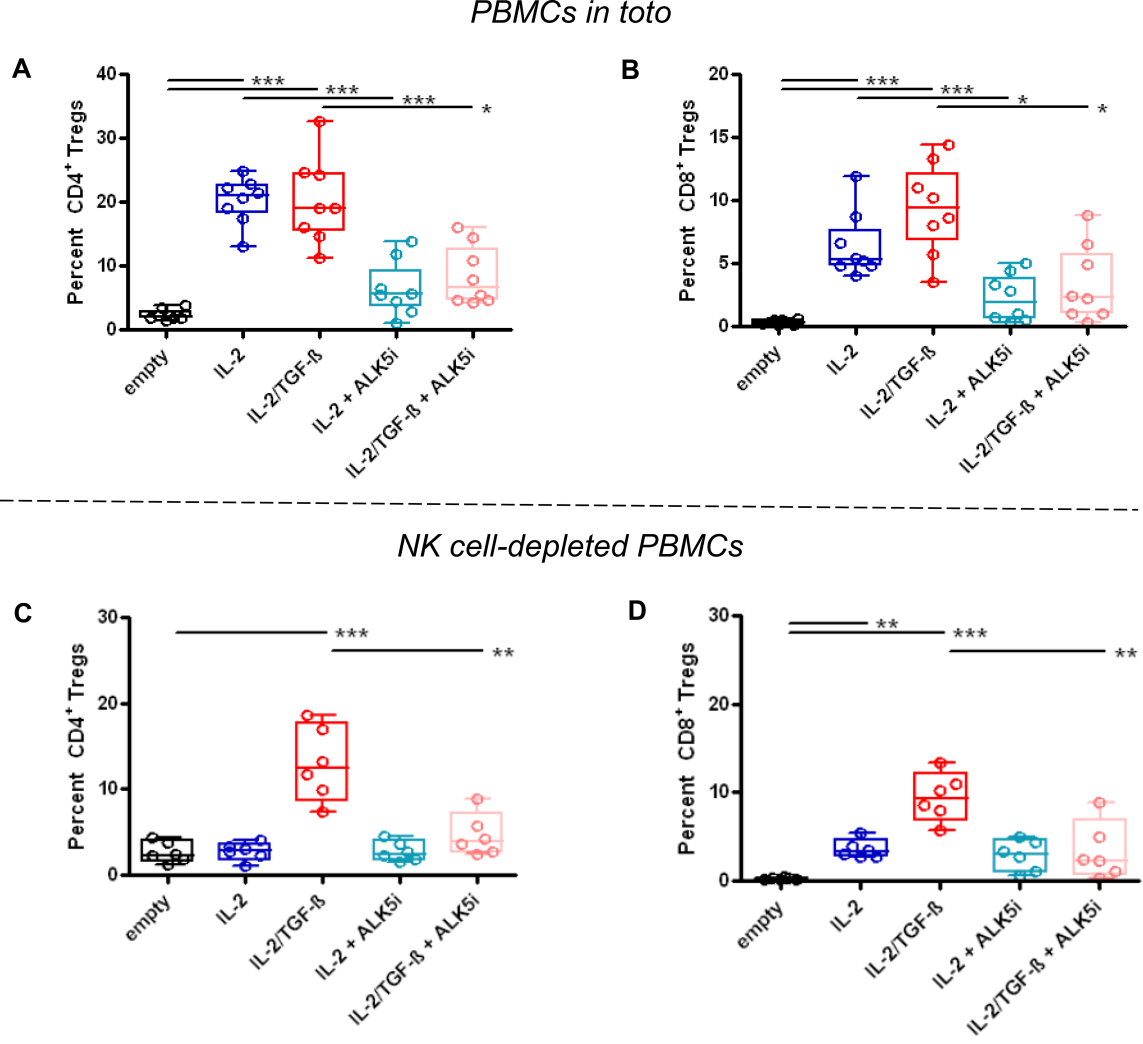
Increased frequency of CD4^+^ and CD8^+^ Tregs in GvHD NSG mice receiving NPs encapsulating IL-2 plus TGF-β or only IL-2. NSG mice received unfractionated **(A, B)** (n=8/group) or NK cell-depleted **(C, D)** (n=6/group) human PBMCs from individual healthy donors together with empty (control) NPs or NPs encapsulating IL-2 (blue) or IL-2/TGF-β (red), alone or together with the TGF-β-signaling inhibitor ALK5i (cyan for IL-2, pink for IL-2/TGF-β), as indicated on the *x* axes. Circulating Tregs among PBMCs (*y* axes) were analyzed *ex vivo* by flow cytometry two weeks after PBMC transfer and NP treatment. *P* *<0.05; **0.005; ***0.0005 vs. empty NPs.

As expected from past work (29, 33, 34), the protective effects of the NPs encapsulating IL-2 and TGF-β associated with increased frequencies of CD4^+^ and CD8^+^ Tregs (**Figures 5A, B**; **Supplementary Figure 1**). NPs containing only IL-2 increased frequencies of CD4^+^ Tregs similarly to those containing both TGF-β and IL-2 (**Figure 5A**), consistently with the concept that NK cells provided TGF-β. Inhibition of TGF-β signaling with ALK5i prevented this increase (**Figure 5A**). The frequency of CD8^+^ Tregs also increased following treatment with NPs encapsulating only IL-2, and this increase was also abrogated by inhibiting TGF-β signaling (**Figure 5B**). Therefore, NPs containing only IL-2 had same effects on induction of CD4^+^ and CD8^+^ Tregs as those with NPs encapsulating both IL-2 and TGF-β, implying a dispensability of TGF-β in the NPs for the induction of CD4^+^ and CD8^+^ Tregs. Although depletion of NK cells abolished the increase of CD4^+^ Tregs induced by NPs encapsulating only IL-2, a moderate decrease of Tregs was seen when using NPs containing IL-2 and TGF-β (**Figure 5C**). Similarly, depletion of NK cells in mice treated with NPs encapsulating only IL-2 also led to a marked reduction in CD8^+^ Tregs frequency (**Figure 5D**). Frequency of these Tregs remained elevated in mice treated with NPs containing IL-2 and TGF-β.

The finding that mice treated with NPs containing TGF-β had accelerated death and yet had circulating Tregs suggests that in the absence of NK cells, induced Tregs were unstable, e.g., possibly converting into effector cells in the GvHD pro-inflammatory milieu. Alternatively, anti-inflammatory cytokines produced by tolerogenic NK cells could inhibit immune cells such as T follicular helper cells and dendritic cells. Future studies will need to investigate these aspects and also address the contribution of non-canonical TGF-β pathways (35) to the observed pro-inflammatory responses linking reduced number of Tregs to an accelerated demise of the mice.

**Figure 6:**
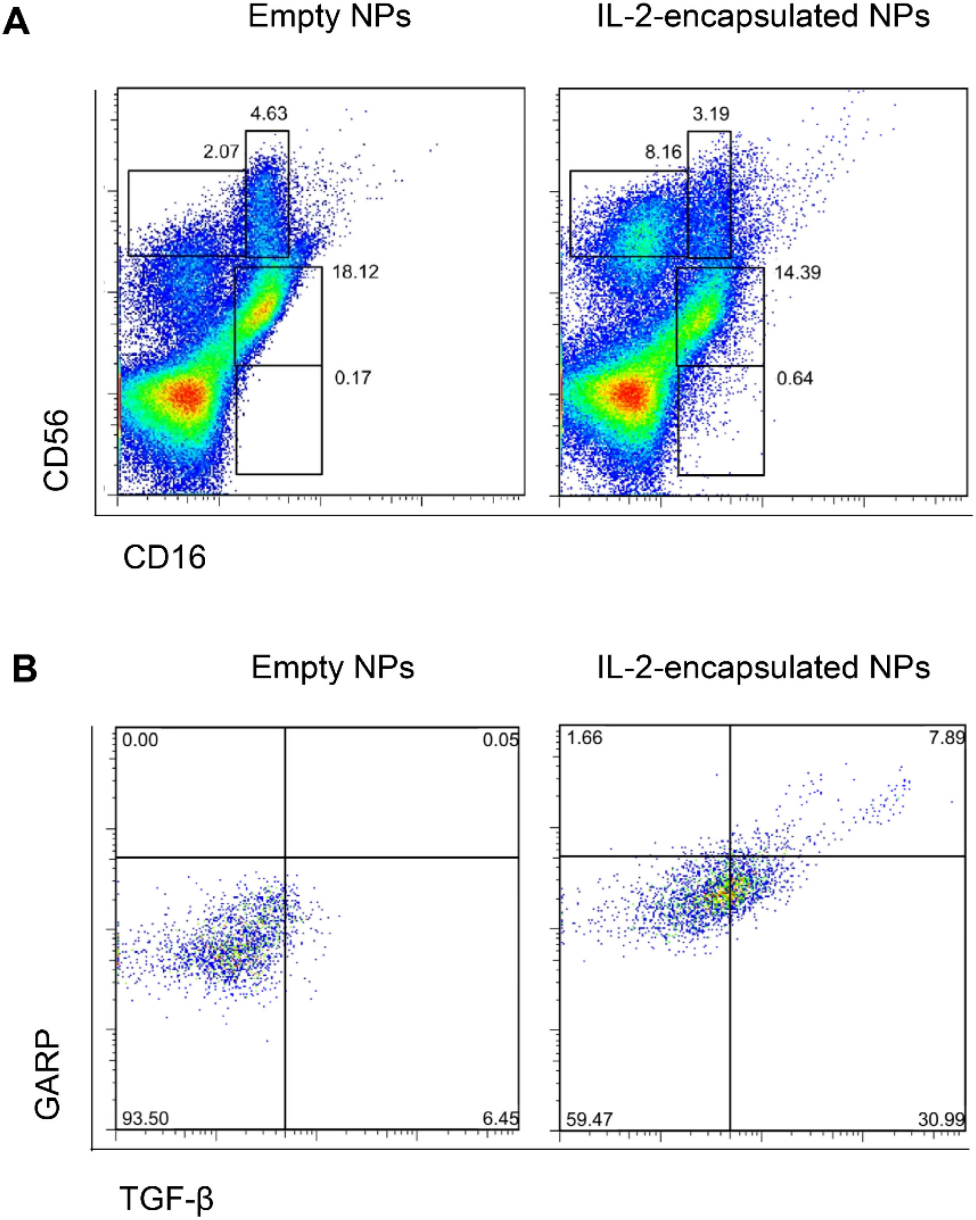
NPs encapsulating IL-2 induce TGF-β-expressing CD56^bright^CD16^-/dim^ NK cells. Human PBMCs were cultured for 5 days in the presence of anti-CD2-coated NPs encapsulating IL-2 or not (empty NPs). Flow cytometry evaluated the expression of CD16, CD56 **(A)**, TGF-β and GARP **(B)**. Representative experiments using five donors and with similar results. **(A)** NK cell populations defined by co-staining with CD56 and CD16. Upper left gate: CD56^bright^CD16^-^ cells; upper right gate: CD56^bright^CD16^dim^ cells; middle gate: CD56^dim^CD16^+^ cells; lower gate: CD56^-^CD16^+^ cells. **(B)** Intracellular expression of TGF-β and surface GARP in CD56^bright^-gated cells from the PBMC cultures with empty NPs or NPs encapsulating IL-2.

Next, we confirmed *in vitro* that NPs decorated with anti-CD2 antibodies encapsulating only IL-2 could induce TGF-β-producing NK cells (**Figure 6**). Induced CD56^bright^CD16^-/dim^ NK cells included a subset of cells co-expressed glycoprotein-A repetition predominant (GARP) (**Figure 6**), a molecule that binds latent TGF-β on the surface of NK cells, facilitating its activation into active TGF-β (36, 37) for the maintenance of Tregs (38).

**Figure 7:**
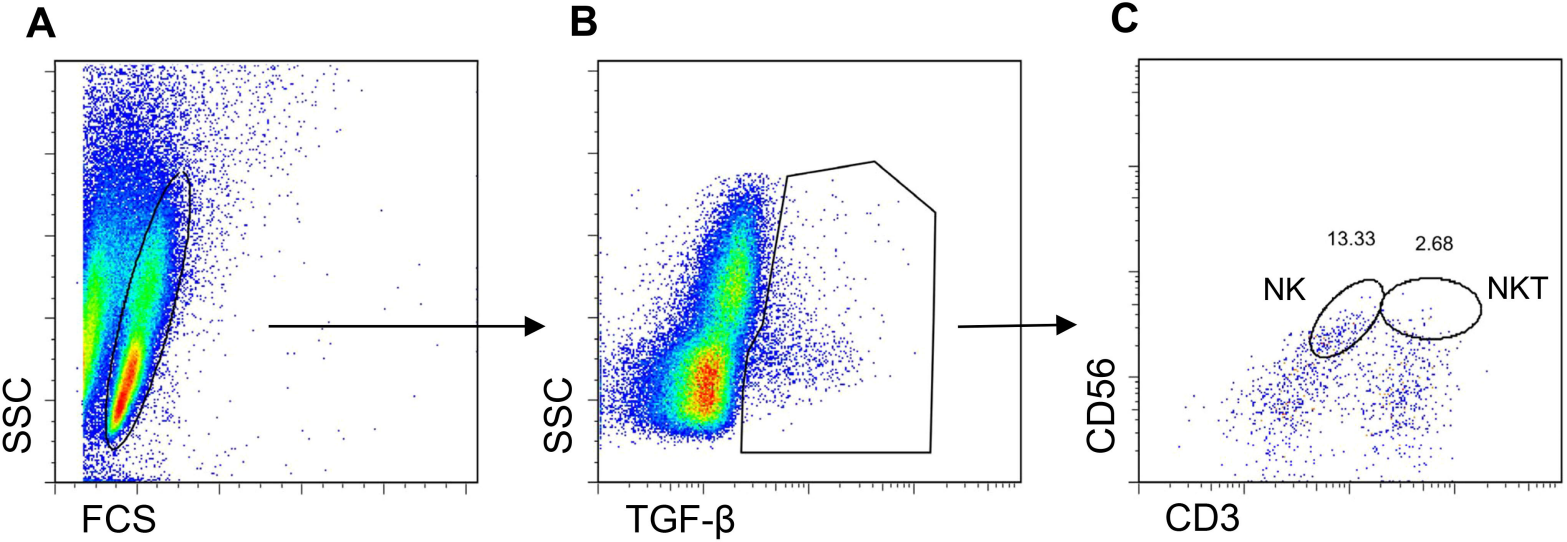
NPs encapsulating IL-2 induce TGF-β-expressing NK cells and NKT cells. Human PBMCs from individual donors were cultured with anti-CD2 antibody-coated NPs encapsulating IL-2. Flow cytometry on day 5 assessed coexpression of CD56 and CD3 together with intracellular TGF-β. **(A)** Gating on PBMCs for the TGF-β-expressing cells **(B)** expressing CD56 and CD3 **(C)** for the identification of TGF-β^+^ NK cells and NKT cells. Representative of five experiments on five donors.

**Figure 8:**
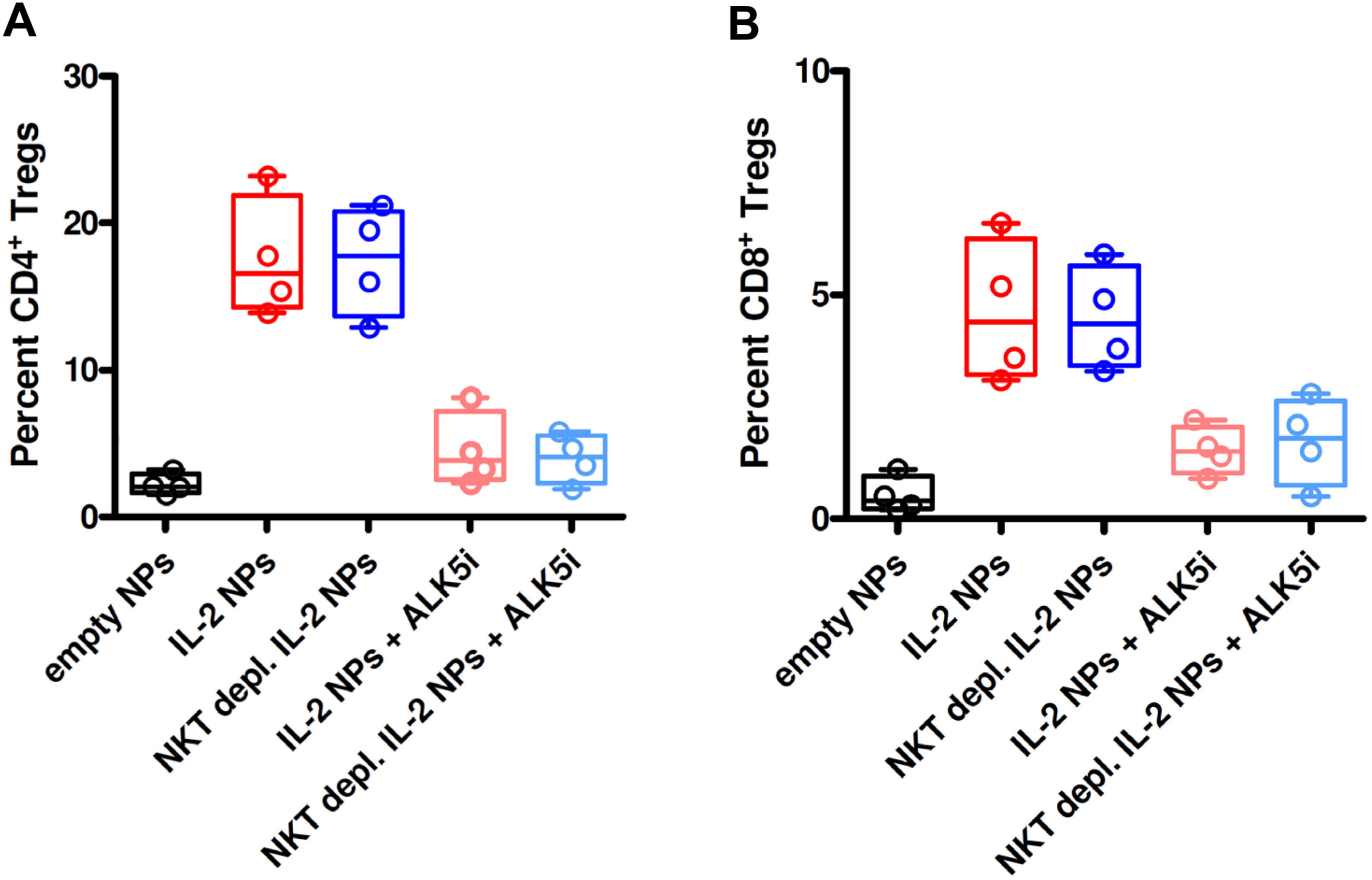
Depletion of NKT cells from PBMCs does not influence the induction of CD4^+^ and CD8^+^ Tregs in GvHD mice treated with NPs encapsulating IL-2. NSG mice (n=4/group) received unfractionated or NKT cell-depleted human PBMCs from individual donors together with empty (control) NPs or anti-CD2 Ab-decorated NPs encapsulating IL-2, alone or together with the TGF-β-signaling inhibitor ALK5i, as indicated on the *x* axes. Circulating CD4^+^ Tregs **(A)** and CD8^+^ Tregs **(B)** were visualized by flow cytometry on peripheral blood two weeks after transfer of the PBMCs and NP treatment. *P ns* in the comparisons between NKT cell-depleted vs. non-depleted.

Since NK cells share markers with NKT cells, both could be producers of TGF-β. However, we found that treatment with CD2-targeted NPs encapsulating IL-2 associated with greater frequencies of TGF-β-producing NK cells as compared to small numbers of TGF-β-producing NKT cells (**Figure 7**; **Supplementary Figure 2**). *In vivo* studies showed that the tolerogenic effects of the CD2-targeted NPs encapsulating only IL-2 depended on NK cells and not on NKT cells, given the lack of differences in the induction of CD4^+^ and CD8^+^ Tregs in the presence or absence of NKT cells (**Figure 8**).

## 4 Discussion

Although it is known that TGF-β is essential for the maintenance and survival of Tregs (8, 39), all cellular sources of this cytokine have not been unequivocally defined. Here we show that the TGF-β produced by tolerogenic NK cells stabilizes newly generated CD4^+^ Tregs and is critical for their sustained effects in a systemic inflammatory disease.

Blockade of TGF-β signaling or depletion of NK cells abolished the therapeutic effects of the tolerogenic NPs in GvHD mice and enhanced severity of the disease. The conversion from a tolerogenic state to an immunogenic one following the blockade of TGF-β signaling in the host suggested that the tolerogenic effects of IL-2 on Tregs depended on TGF-β. NK cell depletion studies showed that the TGF-β required to sustain therapeutic Tregs was provided by NP-induced tolerogenic NK cells, and that without NK cells, the Tregs induced by the NPs were unstable. These findings indicate that tolerogenic NK cells represented the main source of the TGF-β required to stabilize and support newly induced pTregs.

NK cells are a major component of the innate immune system. They express activating receptors that enable the killing of microbe-infected and transformed cells, and inhibitory receptors that prevent them from operating as effector cells. NK cells, including their CD56^bright^ subset, are also important producers of cytokines that influence both T and B cell responses (40, 41). Here we describe TGF-β-producing CD56^bright^ NK cells that provides essential support to stabilizing and maintaining functional Tregs.

While many cell types can produce TGF-β as an inactive precursor (42), NK cells are the only human lymphocyte population that constitutively releases biologically active TGF-β (12). This active TGF-β is increased following engagement of cell surface CD2 on NK cells, and this in turn facilitates induction of CD4^+^ and CD8^+^ Tregs (33).

Others have previously shown that NK cells can produce TGF-β. One group reported that NK cell-derived TGF-β could be a homeostatic regulator of IFN-γ production (43), and another reported that a genetic deficiency of β-cell renalase upregulated NK expression of LAP/TGF-β1, promoting transplant survival (44). Similar to our results, another group reported that enabling NK cells to produce TGF-β could restore self-tolerance to islet cells in non-obese diabetic mice (45).

Our observation that the TGF-β provided by tolerogenic NK cells can stabilize pTregs could help explain the protective effects of NK cells described in several models of autoimmune diseases (46). It is of interest that like our findings in autoimmune disease, TGF-β and NK cells also have important roles in the maternal/fetal tolerance of pregnancy (47, 48). TGF-β promotes the conversion of NK cells that migrate to the maternal/fetal interface to become TGF-β- producing CD56^bright^ NK cells that provide essential support for the decidual Tregs that prevent rejection of the semi allogeneic fetus (49). NK cell removal adversely affects successful pregnancies (50).

In this study we observed the induction of CD8 Tregs as well as CD4 Tregs. The role of CD8 Tregs remains to be identified. Differently from CD4 Tregs, NK cell depletion did not decrease NP-induced expansion of CD8 Tregs. Some authors reported important roles for CD8 Tregs in the prevention of type 1 diabetes in children (51) and in lupus following stem cell transplantation (52). We have reported that human CD8 Tregs induced *ex-vivo* with IL-2 and TGF-β could protect immunodeficient mice from human GVHD (34). We also induced *ex vivo* human CD8 Tregs that could protect immunodeficient mice from human GvHD (53).

Importantly, our previous studies showing that NPs containing only IL-2 could prevent anti-DNA antibody production in mouse lupus suggested that NK cell-derived TGF-β could eliminate the need to encapsulate this cytokine in the NPs for disease protection. The same study showed that dose dependent increase in NK cells appeared to protect mice from developing renal disease, and that this protective effect was also TGF-β dependent (29). We suggest that this increase in tolerogenic NK cells was probably the reason why a short (2 week) course of NPs had the long term effects observed in mouse lupus and GVHD (34). 

The finding that the tolerogenic effects of IL-2 on Tregs are TGF-β dependent and the non-redundant role of the NK-derived TGF-β on CD4^+^ Tregs have special clinical significance in immune-mediated disorders characterized by abnormal IL-2 and/or TGF-β production such as in SLE, where the production of IL-2 and TGF-β is decreased (11, 12). In SLE, NK cells are reduced in number and/or are dysfunctional, particularly during active disease flares (54). Unlike T cells that express IL-2Rα, NK cells preferentially express IL-2Rβ. Because IL-2 muteins are structured to bind only IL-2Rα, they cannot interact with NK cells and to correct defects. This inability could explain why clinical trials in SLE with these agents have failed to meet primary end points (55, 56). Missing was the TGF-β contribution by tolerogenic NK cells.

Also clinically relevant, because TGF-β was produced locally in vivo, it was not necessary to encapsulate this cytokine in the NPs. Because of its pleotropic effects, can have toxic pro-inflammatory activities TGF-β in certain contexts (57).

In summary, this study emphasizes the synergistic effects of IL-2 and TGF-β in the generation of Tregs, extending the known tolerogenic dependence of TGF-β on IL-2 to the converse, where TGF-β production by a population of NK cells is key in supporting Tregs. This study identifies a synergistic activity between innate and adaptive immune response in mechanisms of immune tolerance that can be targeted for the prevention and treatment of chronic immune-mediated diseases. 

Since this article was accepted for publication, the authors have become aware of another report describing suppressive TGF-β producing NK cells. A cluster of TGF-β1high CD56^bright^ NK cells linked to protection from GVHD post hematopoietic stem cell transplantation was described like the NK cells described in this report^1^. These could be induced by IL-2 and TGF-β1 but were unstable. By contrast, the TGF-β producing NK cell we have induced in vivo with CD2 targeted NPs containing IL-2 harvested 5 weeks after NP administration protected lupus mice from renal disease^2^.

## Data Availability

The original contributions presented in the study are included in the article/**Supplementary Material**. Further inquiries can be directed to the corresponding authors.

## Footnotes

1 Mathews JA, Borovsky DT, Reid KT, Murphy JM, Colpitts SJ, Carreira AS, et al. Single cell profiling of hematopoietic stem cell transplant recipients reveals TGF-beta1 and IL-2 confer immunoregulatory functions to NK cells. iScience. (2024) 27(12):111416. doi: 10.1016/j.isci.2024.111416

2 Horwitz DA, Liu A, Bickerton S, Castaldo G, Matarese G, Fahmy TM, et al. Anti-CD2 Antibody-Coated Nanoparticles Containing IL-2 Induce NK Cells That Protect Lupus Mice via a TGF-beta-Dependent Mechanism. Front Immunol. (2020) 11:583338. doi: 10.3389/fimmu.2020.583338
