# Supplementary material for: CD2-targeted nanoparticles encapsulating IL-2 induce tolerogenic Tregs and TGF-β-producing NK cells that stabilize Tregs for long-term therapeutic efficacy in immune-mediated disorders
Source: Front Immunol. 2025 Jul 29;16:1587237. doi: 10.3389/fimmu.2025.1587237 (PMC12339487; doi:10.3389/fimmu.2025.1587237)
Supplement: Supplementary file 1 [file DataSheet1.pdf]

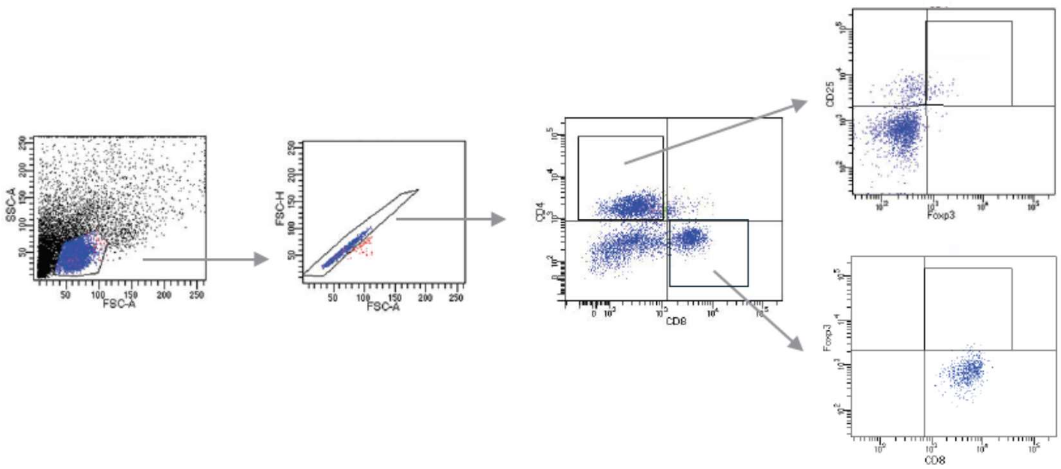

Supplemementary Figure 1. Representative gating strategy for the identification of the Tregs**.**

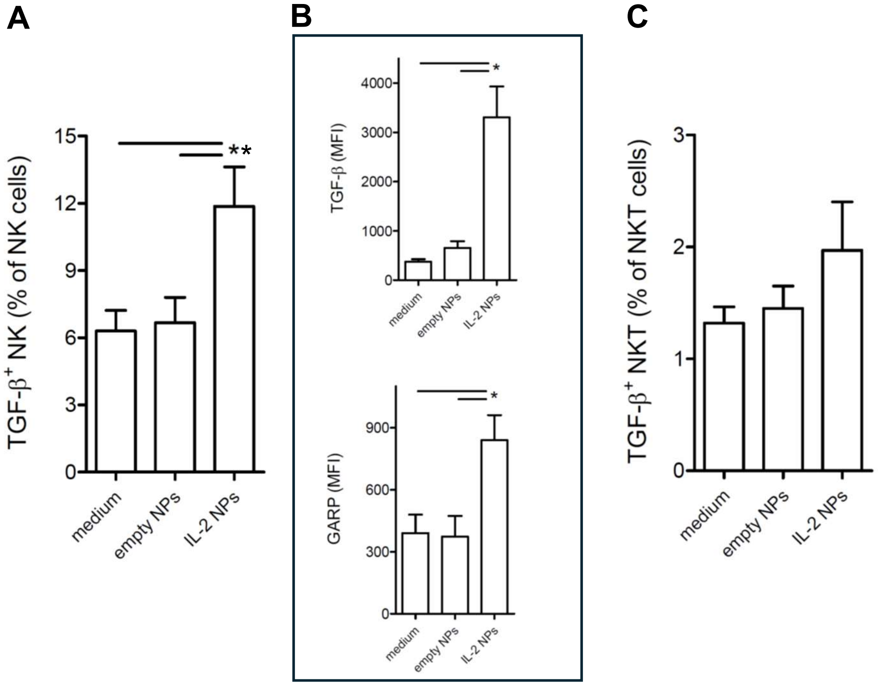

Supplementary Figure 2. CD2-targeted NPs encapsulating IL-2 induce TGF-β-expressing NK cells. Human PBMCs from healthy donors were cultured for 5 days in medium only or with CD2-targeted empty NPs or CD2-targeted NPs encapsulating IL-2. Gating for quantitation of cells was done by flow cytometry by costaining for CD56, CD3, and intracellular TGF-β. Percentages of TGF-β^+ NK cells (A) and NKT cells (n=5) (C); $P<0.01$. MFI of TGF-β and GARP in TGF-β^+ NK cells (n=3) (B); $P<0.05$.
